# Robert Guthrie and the Trials and Tribulations of Newborn Screening

**DOI:** 10.3390/ijns7010005

**Published:** 2021-01-19

**Authors:** Harvey L. Levy

**Affiliations:** 1 Division of Genetics and Genomics, Boston Children’s Hospital, Boston, MA 02115, USA; harvey.levy@childrens.harvard.edu; 2 Department of Pediatrics, Harvard Medical School, Boston, MA 02115, USA

**Keywords:** Guthrie, newborn screening, phenylalanine, filter paper blood specimen, tribulations

## Abstract

Routine newborn screening for many disorders is now so ingrained in newborn care that there is no question about whether it should be done. However, acceptance of newborn screening was not guaranteed when Robert Guthrie introduced it for phenylketonuria (PKU). This article describes the professional and personal story of Guthrie, a physician and microbiologist, who veered from cancer research to a commitment to prevent intellectual disability from PKU. It recounts how Guthrie was able to overcome strong opposition to mandatory screening from prominent physicians and medical societies, so that newborn screening for PKU would be routinely performed throughout the developed world, and would eventually form the basis for the (much more) comprehensive screening conducted today.

## 1. Introduction

A scientific fact, or medical program, once well established, seems obvious. Of course, the earth revolves around the sun, not vice versa. Of course, our planet is round, not flat. Of course, the hands should be washed before any surgical procedure. Of course, evolution is a fact and is largely controlled by natural selection. And, of course, we should screen all newborn infants for phenylketonuria (PKU). But none of these established verities were obvious or generally accepted when first announced. In fact, they were strongly and often bitterly disputed. It is not only of interest but also instructive to recount their beginnings. So it is for newborn screening.

## 2. From Cancer Research to PKU

In the late 1950s Dr. Robert Guthrie, a medical microbiologist conducting cancer research at the Roswell Park Cancer Institute in Buffalo, New York, became active in the local Association for Retarded Children (ARC) as a result of having a son with intellectual disability. At one of the monthly ARC meetings, he invited as the speaker r. Robert Warner, Director of the Children’s Rehabilitation Center at Buffalo Children’s Hospital. At the meeting, Dr. Warner described his work in treating children with developmental disabilities. This was of great interest to Dr. Guthrie and in a later meeting with Dr. Warner, Dr. Guthrie learned about PKU as a biochemical cause of intellectual disability and the recently developed diet that could control the high level of toxic phenylalanine and result in substantial improvement in the very disruptive behavior of these disabled patients [1]. However, Dr. Warner explained, the blood phenylalanine concentration had to be frequently measured so that the diet could be adjusted for optimal therapy and the method available for this measurement, a tube dilution procedure, was laborious and inefficient. Upon hearing this, Dr. Guthrie thought that he might be able to modify a bacterial inhibition assay he was using to screen cancer patients for antimetabolites so that it would be responsive to phenylalanine. In short order he succeeded, so that there was now a simple and much more rapid method for measuring blood phenylalanine in patients with PKU.

## 3. The Idea of Newborn Screening for PKU

At this point, Guthrie began to realize that perhaps he could apply his new bacterial assay to determine if PKU might be the cause of the intellectual disability in children and adults with no diagnosed cause for their handicap. For this, he needed to be at the Buffalo Children’s Hospital, so, in 1958, he transferred his laboratory from cancer research at the Roswell Park Cancer Center to the Children’s Hospital. At first, he used urine, because he thought urine could be more readily obtained than blood to screen intellectually handicapped individuals. Applying filter paper strips of urine to the assay, he found that this method would indeed identify elevated phenylalanine [2]. While he was involved in this work, his wife suddenly received a call from her sister in Minneapolis informing her that the cause of her daughter’s severe intellectual disability and autism was discovered to be PKU. This had a great effect on Guthrie since he had heard that if the dietary treatment began soon after birth, the effects of PKU could be prevented. He also learned that a baby with PKU would have a very high concentration of phenylalanine in the blood very soon after birth. If the PKU in his niece had been detected just after she was born, she would now be normal! His challenge was to find a way to use his bacterial assay to test the blood of all newborn infants for increased phenylalanine. The obvious blood specimen was serum or plasma, which he knew would work in his assay as well as urine, but he also knew that obtaining this type of blood specimen from newborn infants would be far too difficult. He then thought of collecting drops of blood on filter paper, but would whole blood work in his assay? He quickly discovered that strips of filter paper impregnated with whole blood worked very well. Guthrie was not a pediatrician, but he was informed that blood was often collected in capillary tubes for chemical tests from the lanced heels of babies in the nursery, so perhaps drops of blood from the lanced heel could be impregnated into filter paper. Thus was born the idea of newborn screening for PKU.

## 4. The Complicated Birth of Newborn Screening

Guthrie recognized that before the system could be applied to newborn infants, he had to verify that drops of blood could be readily obtained from lanced skin, that the blood could be easily collected onto filter paper, and that the filter paper containing the blood would allow increased phenylalanine concentrations to be reliably detected by his bacterial assay. For this verification, he arranged a trial of the system at a school for those with learning disabilities where there were individuals known to have PKU. The trial showed that all 17 with known PKU were identified by the system, as well as two additional individuals not previously known to have PKU [3].

However, his system had not been examined in real time, i.e., among newborn infants in a hospital nursery. In 1961, he succeeded in obtaining newborn filter paper blood specimens from two hospitals in Jamestown, NY. These specimens worked as expected in his assay. This convinced Guthrie that the system could work, and he then tried to persuade the regional public health laboratories in New York to perform newborn screening using his test. However, none would do so, reasoning that the frequency of PKU, at that time believed to be 1:25,000, was too rare to justify testing all babies. Shortly thereafter, he exhibited his newborn screening test at an annual meeting of the American Public Health Association, which attracted the attention of officials in the U.S. Children’s Bureau and led to their funding Guthrie to conduct a national trial of his newborn screening test. Guthrie realized that the key to having all newborn infants tested for PKU resided in state health departments, so with these funds he set up what he called a “little factory” to package the materials for the tests so that state public health laboratories could easily perform the bacterial assay with the newborn blood specimens sent from the hospitals. He sent an announcement about this to all of the state public health departments in the U.S., inviting each to send a representative from the laboratory to Buffalo to learn the test and obtain the materials to perform it. 

One of the respondents was Dr. Robert MacCready, Director of the Diagnostic Division of the Massachusetts Public Health Laboratory. Dr. MacCready was a physician who had been in private practice before becoming director. He also had had a child with Down syndrome who died shortly after birth so he not only knew about medical conditions but, like Guthrie, had a particular interest in preventing intellectual disability because of his personal experience. Therefore, though he was close to retirement age, he went to Buffalo as the representative from Massachusetts and, along with much younger laboratory technicians, spent 4 days learning to perform the test. Returning to Massachusetts, he set up the test in the microbiology laboratory of the State Laboratory Institute and, as a physician, was able to contact other physicians in the state, including pediatricians and pathologists, persuading them to obtain and submit filter paper blood specimens from the newborn infants in their hospitals. By the fall of 1962, Massachusetts had established the first universal newborn screening program for PKU [4]. Soon, several other states, including Oregon, Ohio, and Maryland, as well as the New York Regional Public Health Laboratory in Buffalo, began PKU screening.

## 5. Tribulations

By late 1962, over 100,000 newborn tests had been performed and all of the laboratories reported that the test worked very well. Guthrie believed that this information was sufficient evidence for publishing the validity of his test to identify PKU by newborn screening. He also knew that for the screening to be accepted by the medical community, a peer reviewed publication would be necessary. So he prepared a manuscript and submitted it to a leading pediatric journal, but it was rejected. Shortly thereafter, he discovered an article in this journal reporting that a comparison of phenylalanine levels in serum measured by a quantitative method and in the filter paper blood specimen measured by the bacterial assay indicated that the assay did not work well due to a much higher estimated concentration of phenylalanine measured by the bacterial assay than that measured in serum; thus, a very high rate of false positive results would be expected in newborn screening [5]. He realized that the journal had accepted this article rather than his, or rather than publishing both studies. An accompanying editorial concluded that it was therefore premature to advocate routine newborn screening [6]. In a responding letter to the editor, MacCready wrote that 50,000 newborns had been satisfactorily screened in Massachusetts with no “unduly” number of false positive results and that 8 were found to have markedly increased phenylalanine and confirmed to have PKU. Subsequently, Guthrie’s report was published in another pediatric journal, but not until there was a delay of 6 months for a committee report to be received and conclude that the assay was satisfactory for newborn screening [7].

At the time that the Guthrie report was published, MacCready published additional letters to the editor in the *New England Journal of Medicine*, *Lancet*, and the *Journal of the American Medical Association*, all reporting that, of 53,000 newborn infants screened by the Guthrie bacterial assay, 9 babies were confirmed to have PKU and were on dietary treatment [8,9,10], and Massachusetts had passed a law mandating newborn screening for PKU. Nevertheless, Guthrie was having great difficulty getting newborn screening for PKU established throughout the U.S. There was vigorous opposition from physicians and medical societies that considered routine screening to be “socialized medicine” and an infringement on the private practice of medicine. MacCready’s published letters were extremely influential in the adoption of newborn screening for PKU by other states. His statement that 9 of 53,000 newborns, one in 6000, were discovered to have PKU was astounding. Here was this totally unexpected incidence, four times greater than the one in 25,000 assumed prevalence of PKU! It turned out that this presumed incidence of one per 6000 was purely fortuitous. The 10th case of PKU was not discovered by screening in Massachusetts until almost the next 100,000 babies were screened. As MacCready once told me, had these 100,000 babies been the first to be screened newborn screening for PKU might never have occurred. 

Opposition to newborn screening for PKU continued. A meeting of consultants to the California State Department of Health concluded that the Guthrie assay required further evaluation and that knowledge of PKU needed to be more complete before screening could be justified [11]. Additional opposition was based on the compulsory nature of newborn screening that was being mandated by state laws that were beginning to appear in late 1963 after the initial law in Massachusetts. In 1964, the House of Delegates of the American Medical Association voted to oppose “legislation requiring compulsory testing” for phenylketonuria [12]. State medical societies universally continued to oppose mandated screening, the single exception being Massachusetts [13]. Even as late as 1967, when most states had established newborn screening for PKU and had passed laws mandating the screening, opposition to compulsory screening from nationally prominent clinicians and investigators was strongly expressed during a meeting sponsored by the March of Dimes [14]. Most prominent among the opposition was Dr. Samuel Bessman, a medical researcher who, for many years, maintained that not only was compulsory newborn screening for PKU unjustified, but that even dietary treatment for PKU was unjustified [15,16,17,18].

## 6. Guthrie and the Acceptance of Newborn Screening

By 1965, however, most newborn infants were being screened for PKU. This was only 4 years after Guthrie had developed the idea of using his bacterial assay for newborn screening and showing that it worked for this purpose. How did such an almost universal medical practice occur so fast? To answer this question, one had to know Robert Guthrie. Like most geniuses, he was not only very intelligent, but driven by a single pursuit based on a discovery. His recognition of what his discovery could accomplish, and his personal story together with his persistent personality (he said that his father was a traveling salesman and that he had “inherited that gene”) made this possible. I first met Dr. Guthrie in Buffalo in 1968 at a meeting he organized among representatives from the first public health laboratories to have adopted newborn screening. These included Oregon, Ohio, and Maryland. Massachusetts was also invited, but Dr. MacCready did not wish to travel and asked me to go. At this time, I was in my second year as a consultant to the Massachusetts Newborn Screening Program. One million newborn infants had been screened for PKU and Guthrie wished to publish a paper describing this and the results. The meeting was primarily to discuss writing the paper (it was never published; Guthrie would much rather talk than write). 

During this 2-day meeting, I formed an opinion of Guthrie that was confirmed over the many years of my association with him. He was extraordinarily kind and considerate, always calm and soft-spoken. No matter the opposition or, at times, even vitriol leveled against him, he never lost his composure or his unwavering focus on newborn screening. In fact, to call his dedication to newborn screening a “focus” does not adequately describe the intensity of his focus. When discussing anything with Guthrie, whether it was generally medicine or metabolic disease or politics (he was an avowed socialist), he would very soon turn the conversation to newborn screening. And when he began this subject, there was no pause. He could go on until he had to stop for the end of the taxi ride or the destination was reached, or it was well past time to go to sleep, or you simply stood up to leave. As Prof. Horst Bickel said in his obituary to Guthrie, “During a lovely car drive along the Rhine and Mosel rivers (when Guthrie had visited Germany to meet on newborn screening in Europe with Bickel and Prof. Otto Thalhammer of Vienna), neither Thalhammer nor I succeeded in changing Bob’s conversation to ancient castles or different German or Austrian wine growing techniques” [19].

His proselytizing for newborn screening had no boundaries. As early as 1961, when he was trying to establish his assay for newborn screening, he began a lifetime collaboration with Prof. Horst Bickel to begin newborn screening in Germany. In fact, he had met Bickel two years before when Bickel was invited to Buffalo to explain his dietary treatment for PKU. At this meeting Bickel had expressed, in passing, the idea of using Guthrie’s assay for newborn screening. In 1962, he and Ms. Susi, his technician, actually went to Germany to assist Bickel in establishing newborn screening in Marburg, Germany—the first spread of newborn screening beyond the U.S. Subsequently, Guthrie and Bickel together met and organized conferences with influential pediatricians throughout Europe and then Japan, New Zealand, Australia, and Brazil to encourage and help establish newborn screening throughout the developed world and even in a few underdeveloped areas.

## 7. Epilogue

During Guthrie’s lifetime, he was able to realize the extension of newborn screening to many metabolic diseases and to endocrinopathies such as congenital hypothyroidism and congenital adrenal hyperplasiaas well as to hemoglobinopathies such as sickle cell disease. Now newborn screening has been extended to cystic fibrosis, severe combined immunodeficiencies (SCID), adrenoleukodystrophy, spinal muscular atrophy (SMA), and several lysosomal storage disorders. All of these benefit from newborn screening by allowing for early therapy that either prevents the clinical consequences or substantially improves outcome. Although Guthrie’s bacterial inhibition assay has been replaced by much more quantitative and efficient methods in most screening programs, all of the tests are performed on the same filter paper blood specimen that Guthrie developed for newborn PKU screening. As Guthrie said, “I have always considered the filter paper blood specimen to be my most important contribution” [20]. Indeed, it is the Guthrie legacy.
